# The Detection and Characterization of QoI-Resistant *Didymella rabiei* Causing Ascochyta Blight of Chickpea in Montana

**DOI:** 10.3389/fpls.2017.01165

**Published:** 2017-06-30

**Authors:** Ayodeji S. Owati, Bright Agindotan, Julie S. Pasche, Mary Burrows

**Affiliations:** ^1^Department of Plant Sciences and Plant Pathology, Montana State University, BozemanMT, United States; ^2^Department of Plant Pathology, North Dakota State University, FargoND, United States

**Keywords:** Ascochyta blight, pyraclostrobin, QoI-fungicide resistance, G143A mutation, hydrolysis probe assay

## Abstract

Ascochyta blight (AB) of pulse crops (chickpea, field pea, and lentils) causes yield loss in Montana, where 1.2 million acres was planted to pulses in 2016. Pyraclostrobin and azoxystrobin, quinone outside inhibitor (QoI) fungicides, have been the choice of farmers for the management of AB in pulses. However, a G143A mutation in the *cytochrome b* gene has been reported to confer resistance to QoI fungicides. A total of 990 isolates of AB-causing fungi were isolated and screened for QoI resistance. Out of these, 10% were isolated from chickpea, 81% were isolated from field peas, and 9% isolated from lentil. These were from a survey of grower’s fields and seed lots (chickpea = 17, field pea = 131, and lentil = 21) from 23 counties in Montana sent to the Regional Pulse Crop Diagnostic Laboratory, Bozeman, MT, United States for testing. Fungicide-resistant *Didymella rabiei* isolates were found in one chickpea seed lot each sent from Daniels, McCone and Valley Counties, MT, from seed produced in 2015 and 2016. Multiple alignment analysis of amino acid sequences showed a missense mutation that replaced the codon for amino acid 143 from GGT to GCT, introducing an amino acid change from glycine to alanine (G143A), which is reported to be associated with QoI resistance. Under greenhouse conditions, disease severity was significantly higher on pyraclostrobin-treated chickpea plants inoculated with QoI-resistant isolates of *D. rabiei* than sensitive isolates (*p*-value = 0.001). This indicates that where resistant isolates are located, fungicide failures may be observed in the field. *D. rabiei*-specific polymerase chain reaction primer sets and hydrolysis probes were developed to efficiently discriminate QoI- sensitive and - resistant isolates.

## Introduction

The production of cool season pulse crops including chickpea (*Cicer arietinum* L.), field pea (*Pisium sativum* L.), and lentil (*Lens culinaris* Medik) in the Northern Great Plains of the United States is rapidly increasing. Montana is the leading producer of field peas and lentil in the United States, where 1.2 million acres were planted to pulses in 2016 (United States Department of Agriculture and National Agriculture Statistics Service, 2016). However, an increase in pulse production is accompanied by potentially yield-limiting diseases. Chief among these diseases is Ascochyta blight (AB). This is a host-specific disease caused by fungal species including *Didymella rabiei* (Kovachevski) v. Arx (anamorph *Ascochyta rabiei* (Pass) Labr) on chickpea, a species complex consisting of *Didymella pisi* (Barilli et al., 2016), *Peyronellaea pinodes*, and *Peyronellaea pinodella* on field pea (Aveskam et al., 2010), and *Didymella lentis* Kaiser, Wang and Rogers (anamorph *A. lentis* Vassiljevsky) on lentil (Barilli et al., 2016). AB can infect crops at all developmental stages and cause over 40–50% yield reduction under conditions suitable for disease development (Mondal et al., 2005; Wise et al., 2011). In faba bean, 90% losses have been reported (Pande et al., 2005; Barilli et al., 2016). Symptoms of AB can develop on foliar and stem parts of the plant and also cause seed rot. AB is seed- and residue-borne. In the field, disease onset is normally post-flowering (growth stage R1) through plant maturity (growth stage R8). “Infected seeds from diseased pods may be small, shrunken or discolored” (Ye et al., 2000; Gossen et al., 2011). In addition to seed as a source of inoculum, *D. rabiei, D. pisi*, and P. *pinodes* also can subsist in the sexual and/or asexual forms (pseudothecia, pycnidia, and perithecia, respectively), producing ascospores and conidia that can provide a source of inoculum for disease epidemics (Tivoli and Banniza, 2007; Chilvers et al., 2009; Wise et al., 2011).

Management of AB requires an integrated approach including the use of certified disease-free seeds, deep seeding depth, crop rotations of at least 3 years, tillage to bury plant debris, fungicide seed treatment to reduce seed transmission, the use of resistant cultivars and foliar fungicides for prevention or treatment of disease symptoms (Gossen and Derksen, 2003; Wise et al., 2011). The use of resistant varieties and cultural practices can reduce AB, however, resistant varieties are not widely available in the Northern Great Plains. Breeding for resistance to AB is challenging in chickpea. This is because this trait is reported to be rare in the genetic resources available for chickpea (Sharma and Ghosh, 2016). In addition, negative genetic correlation has been reported between resistance to AB and other desirable traits. This was illustrated by Lichtenzveig et al. (2002), who pointed out the negative genetic correlation that existed when combining good resistance to AB and early flowering in chickpea. Current studies in the Middle East, North America and Australia are targeted at developing AB-resistant chickpea genotypes, especially resistance to pathotype IV *D. rabiei*, which is considered highly virulent (Bayaa et al., 2004; Imtiaz et al., 2011; Sharma and Ghosh, 2016). Integration of molecular tools and conventional breeding approaches are being used to accelerate introgression of AB-resistance genes in chickpea genotypes (Sharma and Ghosh, 2016). Fungicides are still widely used to achieve an acceptable level of disease control (Davidson and Kimber, 2007; Lonergan et al., 2015). Broad spectrum protectant fungicides (chlorothalonil) are typically applied pre-flowering and can delay the onset of AB; however, once symptoms appear it is imperative for the grower to apply fungicides that provide a high level of control and move beyond the site of application in plant tissues due to canopy closure (Gan et al., 2006; Davidson and Kimber, 2007; Wise et al., 2009; Lonergan et al., 2015). This concern is heightened in chickpea which is more susceptible to AB when compared with field peas and lentils. Thus, fungicides are frequently applied to chickpea fields and sparingly in field peas and lentils. Three registered fungicide classes that provide a premium level of control for the management of AB include succinate dehydrogenase inhibitors (SDHI; FRAC code 7), demethylation inhibitors (DMI; FRAC code 3), and quinone outside inhibitors (QoI; Fungicide Resistance Action Committee [FRAC] code 11) (Burrows, 2013; Lonergan et al., 2015). These classes of fungicides are considered to have high to medium risk of resistance development (Fungicide Resistance Action Committee, 2015, 2016). This concern is elevated by the site-specific mode of action (MOA) of the fungicides, the polycyclic nature of the disease, airborne spores of the AB pathogens, and of the option of sexual reproduction for most species, allowing rapid mutation and allow inheritance by offspring. The polycyclic nature of the disease predisposes growers to repeat fungicide applications as disease severity can increase rapidly when the weather is favorable, particularly with AB of chickpeas (Banniza et al., 2011; Lonergan et al., 2015; Fungicide Resistance Action Committee, 2016).

Currently, of the three classes of fungicide, QoI fungicides are the choice of most pulse growers for pre- and post-infection management of AB in the United States and Canada (Wise et al., 2008; Delgado et al., 2013; Lonergan et al., 2015; Bowness et al., 2016). Prior to 2007, it was the only available fungicide MOA on pulse crops and resistance developed rapidly in North Dakota and Canada (Gossen and Anderson, 2004; Wise et al., 2011; Bowness et al., 2016). In 2012, SDHIs were registered for use and these have largely been released as blends with other fungicide MOAs due to the high risk of resistance development. In addition, grower’s preference of QoI- fungicides for disease control in pulse fields got a boost when the US Environmental Protection Agency (EPA) approved the use of pyraclostrobin (Headline^®^) to benefit plant health on federally issued labels (Agweb, 2009). This plant health benefit of the QoI-fungicide was reported by Dimmock and Gooding (2002) to prolong grain filling in wheat crops. Furthermore, QoI-fungicide was reported to lower transpiration rates and also reduce the rate of senescence in wheat plants (Petit et al., 2012; Mahoney et al., 2014).

This QoI class of fungicide inhibits mitochondrial respiration in the cytochrome bc1 complex (also known as respiratory chain complex III). The cytochrome bc1 complex facilitates electron transfer from ubiquinol to cytochrome c and links this transfer to proton translocation across the bc1 complex membrane via a mechanism called the proton-motive Q cycle (Brandt and Trumpower, 1994), resulting in ATP/energy production. The fungicide binds to the center of the quinone (Qo) site of the cytochrome bc1 complex (complex III) on the positive side of the inner mitochondrial membrane. This causes depletion of adenosine triphosphate (ATP) that ultimately halts spore germination due to energy inadequacy (Grasso et al., 2006a; Wise et al., 2009; Delgado et al., 2013).

Resistance to QoI fungicides has been reported in wheat pathogens such as *Microdochium nivale Blumeria graminis* f. sp. *tritici, Microdochium majus, Ourcosphaerella graminicola*, (Sierotzki et al., 2000; Amand et al., 2003; Walker et al., 2009; Patel et al., 2012), and several other fungal pathogens including *Cercospora sojinia, Colletotrichum graminicola, Alternaria alternata, Botrytis cinerea, Pyricularia grisea, Podosphaera fusca, Pythium aphanidermatum, Pyrenophora teres*, and *Pseudoperonospora cubensis* (Ishii et al., 2001; Gisi et al., 2002; Avila-Adame et al., 2003; Kim et al., 2003; Ma et al., 2003; Sierotzki et al., 2007; Banno et al., 2009; Samuel et al., 2011; Zeng et al., 2015;). In addition, QoI resistance has been reported in *D. rabiei* in North Dakota and Canada (Gossen and Anderson, 2004; Wise et al., 2009; Delgado et al., 2013). “The mechanism of resistance of *D. rabiei* has been attributed to single amino acid replacement in the cytochrome b protein of the *cytochrome bc1* complex” (Delgado et al., 2013). Currently, three amino acid substitutions are found in the cytochrome b protein of fungal plant pathogens that confer different degrees of resistance to QoI fungicides (Grasso et al., 2006a; Delgado et al., 2013). Low levels of resistance are bestowed by a substitution from phenylalanine to leucine at position 129 (F129L) and a substitution from glycine to arginine at position 137 (G137R) while a high level of resistance is conferred by the amino acid change from glycine to alanine at position 143 (G143A). Fungicide insensitivity have been categorized to two types: quantitative and qualitative. With quantitative insensitivity, the pathogen becomes less sensitive to the fungicide, although higher rates of the fungicide are still effective. Qualitative insensitivity predisposes the pathogen to become completely insensitive to the active ingredient and disease control is no longer achieved at recommended field application rates. Insensitivity to the QoI-fungicides has previously been reported to be qualitative (Delgado et al., 2013; Bowness et al., 2016). QoI-resistant *D. rabiei* isolates have been identified in North Dakota and Montana (Wise et al., 2008, 2009), where the mechanism of resistance was identified as the G143A mutation in the former (Delgado et al., 2013). However, in Montana, there has not been a statewide survey to monitor for resistance to QoI in pulse crops. There is an urgent need to develop a robust screening and monitoring strategy for QoI resistance to help prevent the spread of QoI-resistant AB pathogens in the rapidly increasing pulse acreage in Montana. Thus, the objectives of this study were to (1) determine the presence of resistance to QoI fungicides in AB pathogens from chickpea, field pea, and lentil in Montana; (2) determine the mechanism of resistance associated with QoI-resistant isolates; and (3) develop a robust multiplex real-time PCR diagnostic tool for screening and monitoring of QoI resistance.

## Materials and Methods

### A Collection of *D. rabiei, D. pisi*, and *D. lentis* Isolates

Isolates of *D. rabiei, D. pisi*, and *D. lentis* were obtained from four general sources. Most isolates were obtained from chickpea, field pea and lentil seed lots submitted by growers in 23 Montana counties to the Regional Pulse Crop Diagnostic Laboratory (RPCDL) in Bozeman, MT for testing during the 2014, 2015, and 2016 growing seasons (**Table 1**). A second set of isolates were obtained from chickpea and field pea production fields in Montana where QoI fungicides had been applied. Fields containing chickpea and field pea plants with AB symptoms were sampled on a “W” pattern, with samples taken at a set of intervals of approximately 15 m. The third set of isolates were collected from chickpea and field pea plants with AB symptoms sampled by growers and submitted to the Schutter Diagnostic Laboratory, Bozeman, MT. Finally, some isolates were also obtained courtesy of Julie Pasche at North Dakota State University, Fargo, ND, United States.

**Table 1:** Isolates of *Didymella rabiei, D. pisi*, and *D. lentis* were obtained from chickpea, field pea, and lentil seed lots sent by growers to the Regional Pulse Crop Diagnostics Laboratory (RPCDL) in Bozeman, MT, United States for planting during 2014, 2015, and 2016 growing season.

Collection location by county	Number of seed lots sampled	Total number of isolates	Isolates with quinone outside inhibitor (QoI) resistance^a^
Cascade	6	39	0
Chouteau	2	3	0
Daniels	28	235	5
Dawson	7	38	0
Gallatin	5	16	0
Glacier	2	5	0
Hill	7	45	0
Liberty	2	6	0
McCone	24	92	4
Musselshell	1	1	0
Philips	4	9	0
Pondera	5	6	0
Richland	3	16	0
Roosevelt	23	133	0
Sheridan	15	81	0
Teton	2	4	0
Valley	24	213	2
Yellowstone	2	9	0
Toole	1	2	0
Broadwater	1	2	0
Flathead	1	5	0
Blaine	2	15	0
Garfield	2	15	0
Total	169	990	11

*^a^An isolate was considered resistant if it has the G143A mutation in its *cytochrome b* gene.*

For a standard AB seed test, seed (chickpea *n* = 600, field pea = 400, and lentil = 400) were sterilized in a 1% free chlorine solution for 10 min (International Rules for Seed Testing [IRST], 2017). The solution was drained and seeds were air-dried in the biological cabinet for 30 minutes. Dried seeds (*n* = 10 per plate) were plated on potato dextrose agar (PDA) (Alpha Biosciences Inc., Baltimore, MD, United States). Mycelial growth was noticed from the plated seeds after 11 to 14 days incubation at 20°C ± 1°C in the presence of a routine cycle of cool white fluorescent light (12 h light followed by 12 h dark). The presence of AB pathogens was confirmed by viewing the conidia at 40× magnification.

From plants, isolates were also obtained from a single lesion on symptomatic leaves and stems by cutting the tissue into 3- to 4-cm sections. Stem or leaf sections were immersed in 1% NaOCl for 30 s and rinsed for 30 s in sterile distilled water. Sterilized stem or leaf sections were air-dried in a biological cabinet, placed on PDA and incubated under the conditions described above. Confirmation of the pathogen was conducted as previously described. Conidium of individual isolates from infected seed lots (*n* = 5 to 10) and from symptomatic leaves or stems were incubated on PDA under the conditions previously described. To isolate single spores, three pycnidia from a 10 day old culture were dropped into 2 ml screw cap tube (MP Biomedicals) containing five ceramic beads (MP Biomedicals), 300 μl of sterile water and 0.05% (v/v) tween-20. The mixture was homogenized using a Beadbug homogenizer (Benchmark BeadBug Homogenizer, Benchmark Scientific, NJ, United States) for 60 s at 4000 rpm. The supernatant was removed into a clean 1.5 ml eppendorf tube and diluted 100-fold in sterile water. From the diluted suspension, 100 μl was inoculated on fresh PDA plates and incubated at 20°C±1°C under a diurnal regime of cool white fluorescent light (12 h light followed by 12 h dark). Single spores germinated after 3–5 days. Isolates were stored long-term as conidia on sterile filter paper and as mycelia in 15% sterilized glycerol at –80°C (Skoglund et al., 2011).

### Screening of *Didymella* spp. Isolates for QoI Fungicide Resistance Using a Discriminatory Dose

A total of 990 AB causing isolates were screened for QoI resistance. Of these, 10% were from chickpea, 81% from field peas, and 9% from lentil from seed lots (chickpea = 17, field pea = 131, and lentil = 21) submitted to the RPCDL from 23 counties in Montana. Screening of the isolates was conducted using an *in vitro* agar plate assay according to published methods (Wise et al., 2008) with some modifications. Stock solutions of technical grade formulations of pyraclostrobin (99% active; BASF Corporation, Research Triangle Park, NC, United States) were prepared at a concentration of 5 μg/ml and diluted in acetone. Salicylhydroxamic acid (SHAM; Sigma–Aldrich) was dissolved in methanol and added to all fungicide-amended media at a concentration 100 μg/ml. SHAM minimizes the effect of the alternative oxidative pathway that some fungi use to evade QoI fungicide toxicity in *in vitro* fungicide sensitivity assays (Olaya and Köller, 1999; Bartlett et al., 2002; Wise et al., 2008, 2009; Lonergan et al., 2015). *D. rabiei* and other AB pathogens can utilize this alternative pathway in the presence of QoI fungicides. SHAM has been reported to have no side effects on conidial germination (Wise et al., 2008). The 0 μg/ml treatment served as a control and was amended with 100 μg/ml SHAM, 1 ml of acetone, and 1 ml of methanol per liter.

In addition to agar plate assay, isolates from all the three hosts were screened using a mismatch amplification mutation assay PCR (MAMA-PCR) (Delgado et al., 2013). This PCR-based assay was used to detect mutant isolates of *D. rabiei* bearing the A143 allele of the *cytochrome b* gene. Isolates that had a mycelial growth on fungicide amended media that was 70% of the control plate (without pyraclostrobin fungicide) and that also amplified with the MAMA-PCR were selected for total RNA extraction. Only 11 isolates of *Didymella rabiei* met the two criteria. No isolates of *D. pisi* or *D. lentis* met either of the criteria.

### Total RNA Extraction

Selected isolates were cultured on PDA at 22°C for 7 days at 12 h light. Total RNA was isolated from the fungal isolates using the RNeasy Plant Mini kit (QIAGEN) with alterations in the starting process. Fresh fungal mycelium of each isolate (100 mg) from a 7-day old culture was scraped into a 2 mL screw cap tube (MP Biomedicals) containing 450 μL RLC buffer. The mycelium was disrupted using the BeadBug Benchtop homogenizer (Benchmark Scientific, NJ, United States) set at 3500 rpm for 60 s, and centrifuged at 13, 000 *g* for 1 min. About 400 μL lysate was then transferred to a QIAshredder spin column placed in a 1.5 mL collection tube. From this stage onward, the protocol followed the manufacturer’s instructions. Total RNA was quantified using a NanoDrop 2000c at 260 nm (Thermo Scientific, United States) and adjusted to a final concentration of 100 ng/μL.

### Synthesis of Complementary DNA, RT-PCR, and Sequencing

The first-strand complementary DNA (cDNA) was synthesized using a RevertAid-Reverse Transcriptase kit (Thermo scientific). The cDNA was used in a PCR assay to amplify the coding sequence for amino acid codons 127–276 of the *cytb* gene from *D. rabiei*. This region has been reported to have the G143A mutation and other mutations that confer resistance to QoI fungicides (Fraaije et al., 2002; Delgado et al., 2013).

Standard PCR was conducted in a T100 Biorad thermocycler (Bio-Rad Inc.) with Phusion High-Fidelity PCR master mix, 10 *p*mol each of primer (Delgado et al., 2013) and 50 ng of cDNA template in a final volume of 50 μL. The reaction conditions were: 94°C for 5 min, followed by 35 cycles at 94°C for 30 s, 55°C for 1 min and 72°C for 1 min. PCR was terminated with an extension at 72°C for 5 min. PCR products were analyzed on ethidium bromide-stained 1.5% (w/v) agarose gels run in the 1x tris-acetate-EDTA buffer and exposed to UV light to visualize DNA fragments. Isolates with an expected product of 675 bp were purified directly from PCR products using alcohol precipitation. The purified PCR products were sequenced with primer pair used for the amplification (**Table 2**), in both directions (MCLAB DNA sequencing services).

**Table 2:** Primers pairs used for amplification of the *cytochrome b* gene fragment of *Didymella rabiei* and for detecting the G143A mutation.

Primers	Primer sequence (5′–3′)	Annealing temperature	Reference	Primer pair purpose
A99	TATTATGAGAGATGTAAATAATGG	46°C	Delgado et al., 2013	Sequencing of *cytochrome b* gene
A100	CCTAATAATTTATTAGGTATAGATCTTA	46°C	Delgado et al., 2013	Sequencing of *cytochrome b* gene
A243	GCTTTCCTGGGTTACGTTCT	64°C	This study	Multiplex TaqMan PCR
A244	CCAACTCATGGTATAGCACTCAT	64°C	This study	Multiplex TaqMan PCR
A245res	FAM-TGGGCAAATGTCACTATGAGCTGCTACAG-BHQ1	64° C	This study	QoI-resistant probe (A143 allele)
A246sens	Cy5-TGGGCAAATGTCACTATGAGGTGCTACAG-BBQ	64° C	This study	QoI-sensitive probe (G143 allele)

### Effect of G143A Mutation on *D. rabiei* Fungicide Sensitivity on Disease Control

Greenhouse trials were conducted to determine the level of *in vivo* disease control attainable with QoI fungicides against isolates classified as susceptible or resistant to QoI fungicides based on sequencing results. Five QoI-sensitive *D. rabiei* isolates (AR-405, AR-407, AR-419, AR-439, and AR-430) (Wise et al., 2008) and five QoI-resistant isolates (AR-R001 to AR-R005) were included in the trial (**Table 3**). The five QoI-resistant isolates were isolated from a chickpea seed lot submitted to the RPCDL for seed testing, and five QoI-sensitive isolates were from a baseline population (Lonergan et al., 2015). The QoI sensitivity of these five isolates was determined using pyraclostrobin amended PDA, MAMA-PCR, and mutation analysis of their *cytb* gene.

**Table 3:** List of *D. rabiei* isolates used for the *in vivo* assay.

Isolate ID	QoI status	Location by state
AR-R001	Resistant	Montana
AR-R002	Resistant	Montana
AR-R003	Resistant	Montana
AR-R004	Resistant	North Dakota
AR-R005	Resistant	North Dakota
AR 405	Sensitive	Idaho
AR 407	Sensitive	Idaho
AR 439	Sensitive	Washington State
AR 411	Sensitive	Idaho
AR 430	Sensitive	Idaho

The greenhouse experiments were performed following Pasche et al. (2004, 2005) and Wise et al. (2009). Briefly, chickpea seeds (cv. Troy) were obtained from Washington State Crop Improvement Association (WSCIA). Troy is a moderately resistant chickpea cultivar. The seeds were tested free of seed borne AB and were sown at one plant per pot in 80 ml plastic cones filled with a mixture of peat and Sunshine Mix 1 (Sun Gro Horticulture Inc., Bellevue, WA, United States) at ratio 1:1, and grown at 22 ± 2°C. Fourteen days after planting, chickpea plants were treated with commercial formulations of pyraclostrobin (Headline, 2.09 EC; BASF Corporation) at concentrations of 0, 0.1, 1.0, 10, and 100 μg a.i./ml of water. Fungicides were applied to runoff using a CO_2_-powered Generation III Research Track sprayer (DeVries Manufacturing, United States). About 24 h after fungicide application, chickpea plants were inoculated with a conidial suspension obtained from 14 old culture of QoI-resistant and sensitive *D. rabiei* isolates. Within an hour made conidial suspensions were adjusted to a concentration of 3 × 10^5^ conidia/ml and applied to chickpea plants. Inoculum from each isolate was applied to plants using hand-held spray bottles. Chickpea plants were placed in a mist chamber and held at >90% relative humidity for 36 h at a 14 h photoperiod under artificial lighting. After 11 days, disease severity was assessed visually based on the percent leaf area infected of the whole plant (Reddy and Singh, 1984). The experiment was laid out as a randomized completely block design (RCB). Nine replicates (one plant per replicate) were included in each experiment, and the disease severity was calculated for each observational unit. Percent disease control was calculated by: “[1 – (% diseased tissue/% disease on 0 μg/ml control)] × 100” (Wise et al., 2009). Homogeneity of the variances from the two greenhouse experiments was determined by the Levene’s test (Levene, 1960). Data were converted to percent disease control to enhance direct comparisons between QoI-sensitive and resistant isolates at each fungicide concentration and analyzed using the generalized linear mixed-effects model in lm4/ nlme statistical package (R Core Team, 2013).

### Development of a Multiplex Hydrolysis Probe Assay for the Detection of QoI-Resistant (G143A) and QoI-Sensitive *D. rabiei* Isolates

For simultaneous detection and differentiation of the *D. rabiei* G143A mutants from the sensitive isolates, a primer pair (A243 and A244) and two hydrolysis probes (A245res and A246ses) were designed for a multiplex real-time PCR assay to amplify a 92 bp fragment of the *cytb* gene. The 5′ ends of the probes A245res and A246ses were labeled with 6-carboxy fluorescein (FAM) and cyanine 5 (Cy5), while their 3′ ends were labeled with Iowa black-FQ and Iowa black-RQ quencher, respectively. The primers were designed to flank the region of the G143A mutation, while the two fluorogenic dyes enable the multiplex differentiation between resistant and sensitive alleles. To enhance the efficiency of the probes both probes were designed to have *T_m_* values at least 7°C higher (69°C) than that of the primers (62°C), and their GC content was higher than 45%. The multiplex TaqMan assay was optimized in a final volume of 20 μL containing 10 μL of EconoTaq Plus 2x master mix according to manufacturer’s recommendations (Lucigen Corporation, Middleton, WI, United States), 25 pM of each primer (A-243, A-244), 10 pM of each probe (A-245res, A-246sens) and 3 μL of DNA extract. Cycling parameters were 4 min at 94°C, followed by 35 cycles of 15 s at 94°C and 30 s at 64°C and a final extension at 72°C for 5 min completed the PCR. Primers and hydrolysis probes were synthesized by Integrated DNA Technology (IA, United States). The assay was developed, evaluated and analyzed on the Biorad CFX96 real-time PCR detection system (Bio-Rad Laboratories, Inc., Hercules, CA, United States).

In addition, evaluation of the assay efficiency was determined by plotting cycle thresholds for a six times tenfold dilution starting with 1000 ng of DNA obtained from resistant and sensitive isolates against DNA concentration to yield the standard curves. The result from experiments in the multiplex assay was compared to the uniplex assay using tenfold dilutions from 1000 ng to 1 pg DNA extracts from only isolates that contained the G143A mutation.

## Results

**FIGURE 1:**
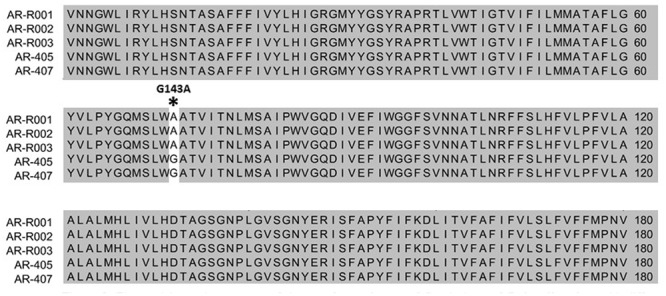
The partial protein sequence of the *cytochrome b* gene of five isolates of *Didymella rabiei* with different Qol sensitivities. Star indicates the G143A amino acid substitution responsible for decreased sensitivity to Qol fungicides. Dark gray highlighted areas represent 100% identities.

From the screening, only 11 isolates of *D. rabiei* amplified with the MAMA-PCR and also had a mycelial growth on fungicide amended media that was 70% of the control plate (without pyraclostrobin fungicide). Multiple alignment analysis of amino acid sequences of the *cytb* gene of the detected QoI-resistant *D. rabiei* isolates showed a mutation that replaced the codon for amino acid 143 from GGT to GCT, resulting in an amino acid change from glycine to alanine (G143A) (**Figure 1**). Other known mutations such as (F129L) and (G137R) were not found in the protein sequences of our QoI-resistant isolates. However, none of the isolates of *D. pisi* and *D. lentis* amplified using MAMA-PCR.

### Effect of the G143A Mutation on *D. rabiei* Fungicide Sensitivity

**FIGURE 2:**
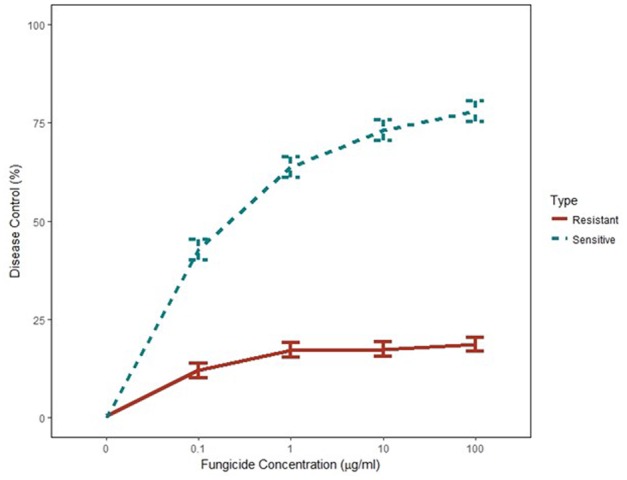
Mean *in vivo* percent disease control for five Qol-sensitive and five Qol-resistant *Didymella rabiei* isolates to pyraclostrobin fungicide concentration (μg/ml). Values include standard errors of disease control measurements obtained from one plant across nine replications in two experiments.

Independent analysis of greenhouse disease control experiments showed that variances were homogeneous and the two experiments were combined for further analysis (*p*-value = 0.05). Disease severity was significantly higher on chickpea plants inoculated with G143A mutant isolates at all concentrations of pyraclostrobin. Percent disease control from the non-treated was calculated to directly compare the two isolate groups. Disease control of G143A mutant isolates was significantly reduced in the pyraclostrobin treatments when compared to wild type isolates at all fungicide concentrations (*p*-value <0.001) (**Figure 2**). About 75% disease control was observed at 10 and 100 μg/ml in wild-type isolates while <25% disease control was observed in G143A mutant isolates.

### Detection of QoI-Resistant (G143A) and QoI-Sensitive *D. rabiei* Isolates Using a Multiplex Hydrolysis Probe Assay

**FIGURE 3:**
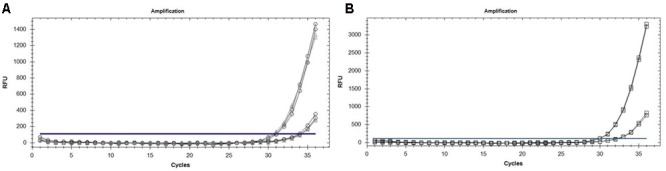
**(A)** Amplification curves showing the detection efficiency of sensitive (○) and resistant (△) alleles using the mixtures of DNA from Qol-resistant and Qol-sensitive isolates as a template. **(B)** Amplification curves showing the detection efficiency of resistant (□) alleles in uniplex TaqMan real-time PCR using DNA from Qol-resistant isolates as a template.

**FIGURE 4:**
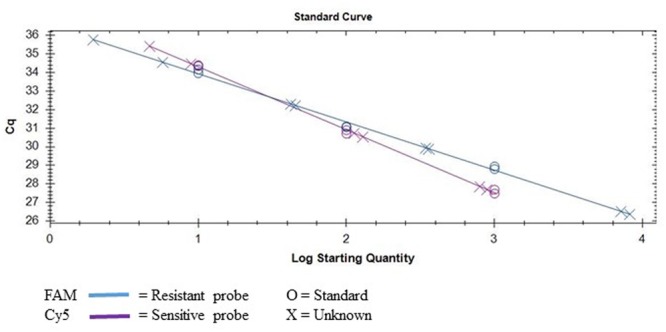
Standard curve obtained by using the multiplex SNP TaqMan assay to detect the G143A mutation in *D. rabiei* isolates collected from Montana.

By deploying the single nucleotide polymorphism (SNP) hydrolysis probe assay, it was possible to detect the G143A mutation and discriminate between QoI- resistant and QoI- sensitive isolates. The SNP could be distinguished using specific probes in which a nucleotide proximal to the 3′ is complementary to one allele but forms as a mismatch with the second allele. The annealing temperature was validated in a temperature gradient assay, the optimum annealing temperature was 64°C for both multiplex and uniplex (data not shown). The optimum concentration of primers and probes that gave the highest reporter fluorescence and the lowest threshold cycle was 20 and 10 μM, respectively, in both tests. To confirm the assay can simultaneously detect the two alleles, DNA from wild-type (QoI-sensitive) and G143A mutant (QoI-resistant) isolates were mixed in the same proportion. Satisfactory discrimination was achieved between the two alleles (**Figure 3**). This emphasized the accuracy of this assay, by its capacity to detect either of the alleles both in a multiplex or uniplex assays, respectively. Standard curves were constructed based on the tenfold dilution series of the wild-type and mutant isolates (**Figure 4**). There was linearity in the amplification across the DNA dilutions and correlation coefficients for the standard curve of the DNAs from wild types and G143A mutant isolates were 0.998 and 0.991, respectively (**Table 4**). The y-axis on the amplification plot measures the relative fluorescence units (RFU), a measure of the amplified DNA, while cycling threshold value (cq) on the x-axis, is inversely proportional to the initial concentration of nucleic acid template in each sample, which correlates to the number of copies in each sample.

**Table 4:** Slope, efficiencies, correlation coefficients (*R*^2^), and *y*-intercepts from amplification of serial dilutions of DNA from *Didymella rabiei* isolates sensitive and resistant to quinone outside inhibitor fungicides using TaqMan single-nucleotide polymorphism assay to detect G143A mutation.

Allele	Slope	Efficiency (%)	*R*^2^	*y*-intercepts
Wild-type-sensitive	3.39	97.2	0.998	37.705
Mutant-resistant	2.59	142.7	0.991	36.531

## Discussion

According to our findings, the G143A mutation is responsible for QoI fungicide resistance in *D. rabiei* isolates from Montana chickpea fields. The gene structure of the *cytochrome b* gene of *D. rabiei* appears to be favorable for the development of a SNP associated with QoI resistance at codon 143 (Delgado et al., 2013). In contrast, the G143A mutation has not been reported in fungal species that have an intron downstream of codon 143 (Grasso et al., 2006b; Sierotzki et al., 2007; Banno et al., 2009; Samuel et al., 2011; Delgado et al., 2013; Zeng et al., 2015). Thus, there is no reported case of QoI resistance in this type of *cytochrome b* gene structure. For example, fungal species such as *A. solani* do not show a G143A mutation due to the lethal effect of proximal exonic flanking sequences on the 5′-splice (Lambowitz and Belfort, 1993; Pasche et al., 2005; Grasso et al., 2006b; Sierotzki et al., 2007; Banno et al., 2009; Delgado et al., 2013). However, G143A mutation has been reported to be responsible for QoI fungicide resistance in *C. sojina*, the causal organism of frog eye leaf spot of soybean (Zeng et al., 2015) and in *B. cinerea*, the causal organism of gray mold (Samuel et al., 2011). In these two organisms, the mutation does not occur in the 5′-splicing site. QoI fungicides do not control fungi bearing the G143A mutation, while those containing the F129L and G137R are controlled to some degree, but at a lower level than wildtype isolates. Similar results were obtained in a study in North Dakota (Wise et al., 2009; Delgado et al., 2013), where the mechanism of resistance of QoI-resistant *D. rabiei* isolates was reported to be a G143A mutation. Noticeably, all the three counties where QoI-resistant *D. rabiei* isolates were found are near North Dakota indicating the QoI-resistant isolates detected could have either spread from North Dakota via seed or were selected for in Montana chickpea fields.

Quinone outside inhibitor fungicide resistance was observed in isolates of *D. rabiei* collected in Montana during the 2012 and 2015 growing seasons (M. Burrows, Personal communication). Prior to this, QoI-resistant isolates were reported in 2009 from chickpea fields in North Dakota (Wise et al., 2009) and have been largely maintained in the population (Delgado et al., 2013). From both seed testing results and the current in-field survey of Montana pulse fields, the frequency of resistant isolates is thus far low (B. Agindotan, personal communication, March 2016). This is different from the report of high frequency of QoI-resistant isolates in North Dakota (Wise et al., 2009; Delgado et al., 2013). This may be due to the comparatively low relative humidity and reduced disease pressure in Montana vs. North Dakota. The relative humidity level in North Dakota is 12.1% higher than Montana in chickpea growing areas (ClimaTemps, 2015). Though this study was targeted at AB of chickpea, field pea, and lentil, only 11 isolates of *D. rabiei* from three seed lots from a larger study of 88 isolates from 17 seed lots were confirmed to be resistant to pyraclostrobin. All isolates contained the G143A mutation which confers resistance to all QoI fungicides (**Table 5**). The frequency of QoI resistance might be on the rise in *D. rabiei* because, from observations during the 2016 crop year, USDA-NASS statistics and grower testimonies, there is increasing chickpea acreage and multiple applications of fungicides to ward off potential fungal attacks. Many of these applications were QoI products solely and in combination with either chlorothalonil or SDHI fungicides such as fluxapyroxad. The proportion of QoI fungicide applied solely is higher than that applied in combination with other fungicides. However, continuation of the practice of applying multiple applications of fungicides including high-risk products such as QoI and SDHI fungicides will select for resistance development. These active ingredients are available as seed treatments and foliar products. Although education is underway, fungicide decisions are often driven by the price and efficacy of the product more than the MOA.

**Table 5:** Distribution of Ascochyta isolates collected per crop in 2014–2016 from Montana.

Crop	Number of Counties sampled	Total Number of seed lots	Total number of isolates	Isolates with QoI resistance
Chickpea	9	17	88	11
Field pea	17	131	810	0
Lentil	7	21	92	0
Total		169	990	11

In contrast with chickpea, field pea and lentil fields are rarely treated with fungicides. This is due to the low foliar disease occurrence in Montana to date. Since seed testing was started in Montana in 2000, the percent of seed lots with at least one seed of 500 infected by AB has increased from 0% (2000–2002) to as high as 25% through 2009. Since the 2010 crop year, the level has increased to 60%. It was 80% in 2017. To date, we have very rarely observed QoI resistance in AB pathogens recovered from a field pea seed lots and never observed QoI resistance in AB recovered from lentil seed lots. This correlates with the low frequency of fungicide application in these crops.

Differences in disease control were observed when QoI-resistant and QoI-sensitive *D. rabiei* isolates were inoculated on pyraclostrobin-treated chickpea plants. Applications of pyraclostrobin at a concentration of 100 μg/ml provided less than 25% control of disease on chickpea plants infected with QoI-resistant isolates. This amount of control is unacceptable in field production. Disease severity of AB was higher on fungicide-treated chickpea plants inoculated with QoI-resistant *D. rabiei* isolates that on plants inoculated with QoI-sensitive isolates study. Several studies have not observed a fitness cost associated with G143A substitution in *cytb* in fungal pathogens (Chin et al., 2001; Avila-Adame et al., 2003; Karaoglanidis et al., 2011; Veloukas et al., 2014). However, once established, resistance is likely to be preserved in the population due to the selective advantage if QoI fungicides continue to be applied. This lack of disease control in QoI-resistant isolates was confirmed a report from North Dakota (Wise et al., 2009). In that study, <50% disease control was achieved with the applications of 100 μg a.i/ml pyraclostrobin to chickpea plants inoculated with QoI-resistant isolates. Considering this, monitoring of QoI resistance in AB pathogens infecting pulse crops is important to prevent the establishment of resistant populations. Due to the widespread occurrence of AB pathogens in seed lots in Montana, favorable environmental conditions will likely lead to a widespread epidemic of the pathogen. Frequent fungicide applications in those circumstances will lead to the establishment of resistant isolates and then additional management strategies will need to be used more effectively to manage fungicide resistant AB pathogens. The risk of fungicide resistance development can be lowered by limiting the number of applications of single-site fungicides with the same MOA, rotation among fungicides with different biochemical modes of action or by using blends of fungicides with different modes of action. This approach was very effective in the control of fungicide-resistant strains of sclerotinia dollar spot and pythium blight in turfgrass (Couch, 2002, 2003).

The development of QoI resistant isolates in the epicenter of pulse production in Montana could cause significant problems for the industry, which in 2016 occupied 1.2 million acres and was valued at $322 million dollars (United States Department of Agriculture and National Agriculture Statistics Service, 2016). To contain the spread of QoI- resistance, monitoring is key. PCR-based tools like the one developed in this study are needed to monitor the shift in fungicide sensitivity of field population of fungal pathogens. Furthermore, integrated pest management practices including crop rotation, tillage to bury infested residue, and host resistance (where available) can help reduce the risk of fungicide resistance (Zeng et al., 2015). There should be caution in the application of foliar fungicides for reasons other than disease control including ‘plant health benefit.’ Other practices to prevent fungicide resistance development include restricting the number of QoI fungicide applications to two to four per season as specified on the label, using QoIs preventatively rather than after disease has developed, not allowing sequential applications of QoI products, as specified on the label, rotating fungicide modes of action when multiple applications are necessary, using pre-mixtures or tank mixtures different modes of action, and always applying the recommended labeled rate (Kitchen et al., 2016). The minimum recommended rates of each fungicide in the tank mix should be used (Damicone and Smith, 2009). Failure to practice these guidelines will apply additional selection pressure on *D. rabiei* and other fungal pathogens which will consequentially result in fungicide resistance development.

Various polymerase chain reaction- based methods such as PCR-RFLP and CAPS methods have been developed for the detection of G143A mutation. However, limited quantitative methods such as allele-specific real-time PCR have been developed for a few pathogens (Samuel et al., 2011; Zeng et al., 2015). The real-time PCR assay enhances high throughput and accuracy during QoI-sensitivity screening of isolates when compared to the conventional *in vitro* test using fungicide amended PDA plates. This test takes about 7 to 10 days to get a result, in addition to the logistics required to screen multiple isolates. The hydrolysis probes were designed to have *T_m_* values at least 7°C higher (69°C) than that of the primers (62°C), and their GC content was higher than 45%, improving the specificity of the assay in discriminating sensitive from resistant alleles. Furthermore, modification of the primers and probes avails this assay the potential to monitor G143A mutation in *D. pisi, D. lentis*, and *A. alternata*, thus it can serve as a tool to monitor QoI resistance in other fungal pathogens and also for routine screening of fungal isolates in diagnostic laboratories. In addition, this technique is suitable for future research targeted at determining modification in the frequency of G143 and A143 alleles and also to determine the fitness of QoI-resistant and sensitive isolates of fungal pathogens.

## Conclusion

This study was successful in detecting the presence of QoI- resistant *D. rabiei* isolates, characterizing the mechanism of resistance and developing a diagnostic tool for QoI resistance in *D. rabiei* that will allow high throughput and accurate screening of G143A mutants. Other researchers have been successful in developing a molecular technique to detect the G143A mutation (Delgado et al., 2013). This is a qualitative assay that cannot be used to monitor the frequency of G143 and A143 alleles both in individual isolates and in mixed field populations. The assay reported in this study streamlines the detection process. This process could be used for large-scale surveys, as well as rapid identification of insensitivity to QoI fungicides. Furthermore, this technique can be used in studies to determine changes in the frequency of G143 and A143 alleles in *D. rabiei*.

## Author Contributions

AO and MB designed the greenhouse experiments. AO carried out the greenhouse experiments. Isolates of *D. rabiei* were donated by JP. AO and BA designed the hydrolysis probe assay experiment. AO carried out the assay development. AO conducted out most of the analysis and wrote the manuscript. The manuscript was reviewed by MB, BA, and JP.

## Conflict of Interest Statement

The authors declare that the research was conducted in the absence of any commercial or financial relationships that could be construed as a potential conflict of interest.
